# Supplementary figures and images for: Insects evolved a monomeric histone-fold domain in the CENP-T protein family
Source: EMBO Rep. 2025 Oct 29;26(23):5799–825. doi: 10.1038/s44319-025-00603-5 (PMC12678787; doi:10.1038/s44319-025-00603-5)

Source data Figure 1B (uncropped blot)

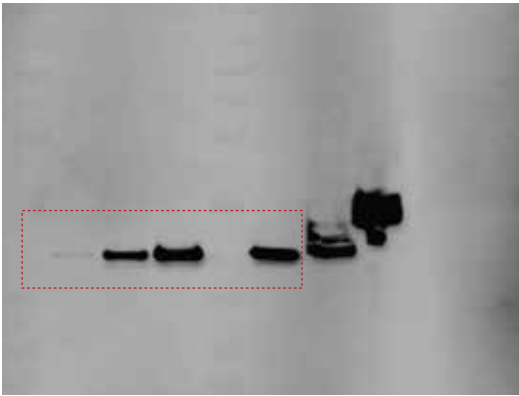

Supplement: Supplementary file 4 — Source data Fig. 1 [file 44319_2025_603_MOESM4_ESM.zip › Figure 1/FIgure 1B/Figure 1B-uncropped blot.pdf]
