# Supplementary material for: Insects evolved a monomeric histone-fold domain in the CENP-T protein family
Source: EMBO Rep. 2025 Oct 29;26(23):5799–825. doi: 10.1038/s44319-025-00603-5 (PMC12678787; doi:10.1038/s44319-025-00603-5)
Supplement: Supplementary file 5 — Source data Fig. 2 [file 44319_2025_603_MOESM5_ESM.zip › Figure 2/Figure 2E/Dataset EV1-crosslink_visualization.pdf]

#Custom script used to visualize protein crosslinks in 1-D

```
import matplotlib.pyplot as plt
import matplotlib.patches as patches
import numpy as np

def plot_crosslinked_protein(protein_sequence, crosslinks,
special_crosslinks=None, arc_color='darkgray', arc_thickness=1,
save_path=None):
    if special_crosslinks is None:
        special_crosslinks = []

    protein_length = len(protein_sequence)
    fig, ax = plt.subplots(figsize=(15, 6))

    # Colored segments (1-based indexing)
    highlight_regions = [
        (19, 33, 'lightblue'),
        (36, 57, 'lightblue'),
        (63, 75, 'lightblue'),
        (82, 92, 'lightgrey'),
        (95, 100, 'lightgrey')
    ]
    for start, end, color in highlight_regions:
        width = end - start + 1
        patch = patches.Rectangle((start, 1), width, 2,
facecolor=color, edgecolor='none', zorder=1)
        ax.add_patch(patch)

    # Draw the outer protein box on top
    protein_outline = patches.Rectangle((1, 1), protein_length, 2,
edgecolor='black', facecolor='none', linewidth=1.5, zorder=2)
    ax.add_patch(protein_outline)

    # Vertical lines for K (blue), D/E (red) – 1-based indexing
    for i, aa in enumerate(protein_sequence):
        x_pos = i + 1
        if aa == 'K':
            ax.plot([x_pos, x_pos], [1, 3], color='blue', linewidth=1)
        elif aa in ['D', 'E']:
            ax.plot([x_pos, x_pos], [1, 3], color='red', linewidth=1)

    # Draw arcs for crosslinks (start from top of box)
    for link in crosslinks:
        start, end = sorted(link)
        mid = (start + end) / 2
        radius = (end - start) / 2
        theta = np.linspace(0, np.pi, 100)
        x = mid + radius * np.cos(theta)
        y = 3 + radius * np.sin(theta)

        color = 'black' if link in special_crosslinks else arc_color
        ax.plot(x, y, color=color, linewidth=arc_thickness)

    # Tick marks and labels (1-based indexing)
    tick_line_y = 0.8
    for i in range(1, protein_length + 1):
        if i % 10 == 0:
            ax.plot([i, i], [0.9, tick_line_y], color='black',
```

```
linewidth=1)
            ax.text(i, tick_line_y - 0.1, f'{i}', ha='center',
va='top', fontsize=8)
        else:
            ax.plot([i, i], [0.95, tick_line_y], color='black',
linewidth=0.5)

    ax.set_xlim(-5, protein_length + 5)
    ax.set_ylim(0, max([abs(link[1] - link[0]) for link in
crosslinks]) / 2 + 5)
    ax.set_aspect('equal')
    ax.axis('off')
    plt.title("Protein Crosslinking Visualization")

    if save_path:
        plt.savefig(save_path, format='svg')

    plt.show()

# Example usage
protein_sequence =
"MKYKPPKRYQPKNASWTTKRLYKYLEDKLEPKYDYKARVRAEKLVETIYHFTKEVKKHEVAPNDAVDVL
KHEMARLDIVKTHFDFYQFFHDFMPREIRVKVVPDIVNKITIPRNGVFSEILSGHAVHA "  #
Example amino acid sequence
crosslinks = [(32, 46), (110, 64), (30, 57), (56, 59), (104, 57),
(100, 59), (32, 42), (27, 57), (28, 59), (52, 46), (30, 47), (26, 57),
(30, 56), (108, 67), (27, 56), (28, 46), (49, 46), (28, 54), (30, 43),
(110, 67), (30, 49), (26, 56), (27, 47), (56, 64), (32, 54), (47, 46),
(100, 64), (24, 59), (26, 47), (28, 64), (36, 59), (35, 59), (28, 42),
(43, 64), (27, 43), (108, 59), (100, 67), (122, 64), (30, 52), (33,
59), (28, 67), (27, 49), (110, 59), (128, 70), (24, 54), (30, 53),
(128, 67), (32, 59), (24, 67), (119, 80), (128, 72), (56, 67), (26,
43), (128, 59), (24, 64), (128, 57), (26, 49), (122, 54), (128, 54),
(118, 77), (128, 64), (104, 86), (64, 1), (59, 1), (24, 46), (122,
77), (128, 80), (128, 32), (100, 84), (122, 84), (59, 5), (64, 5),
(27, 52), (128, 33), (96, 86), (128, 81), (128, 52), (128, 53), (27,
53), (128, 34), (118, 91), (122, 34), (67, 1), (26, 52), (26, 53),
(128, 35), (128, 77), (110, 104), (122, 30), (128, 84), (128, 30),
(67, 5), (24, 42), (43, 67), (108, 64)]  # Example crosslinks
special_crosslinks = [(100,59), (100,64), (100,67), (100,84),
(96,86) ]  # Example special crosslinks with different color
arc_color = 'darkgray'
arc_thickness = 1.25
save_path = '/Users/ssankara/Documents/bmT_180425_2.svg'  # Save path
for the SVG file

plot_crosslinked_protein(protein_sequence, crosslinks,
special_crosslinks, arc_color='darkgray', arc_thickness=arc_thickness,
save_path=save_path)
```
