# Supplementary material for: Insects evolved a monomeric histone-fold domain in the CENP-T protein family
Source: EMBO Rep. 2025 Oct 29;26(23):5799–825. doi: 10.1038/s44319-025-00603-5 (PMC12678787; doi:10.1038/s44319-025-00603-5)
Supplement: Supplementary file 5 — Source data Fig. 2 [file 44319_2025_603_MOESM5_ESM.zip › Figure 2/Figure 2E/Dataset EV1_crosslink_visualization.rtf]

#Custom script used to visualize protein crosslinks in 1-Dimport matplotlib.pyplot as pltimport matplotlib.patches as patchesimport numpy as np def plot_crosslinked_protein(protein_sequence, crosslinks, special_crosslinks=None, arc_color='darkgray', arc_thickness=1, save_path=None):    if special_crosslinks is None:        special_crosslinks = []     protein_length = len(protein_sequence)    fig, ax = plt.subplots(figsize=(15, 6))     # Colored segments (1-based indexing)    highlight_regions = [        (19, 33, 'lightblue'),        (36, 57, 'lightblue'),        (63, 75, 'lightblue'),        (82, 92, 'lightgrey'),        (95, 100, 'lightgrey')    ]    for start, end, color in highlight_regions:        width = end - start + 1        patch = patches.Rectangle((start, 1), width, 2, facecolor=color, edgecolor='none', zorder=1)        ax.add_patch(patch)     # Draw the outer protein box on top    protein_outline = patches.Rectangle((1, 1), protein_length, 2, edgecolor='black', facecolor='none', linewidth=1.5, zorder=2)    ax.add_patch(protein_outline)     # Vertical lines for K (blue), D/E (red) – 1-based indexing    for i, aa in enumerate(protein_sequence):        x_pos = i + 1        if aa == 'K':            ax.plot([x_pos, x_pos], [1, 3], color='blue', linewidth=1)        elif aa in ['D', 'E']:            ax.plot([x_pos, x_pos], [1, 3], color='red', linewidth=1)     # Draw arcs for crosslinks (start from top of box)    for link in crosslinks:        start, end = sorted(link)        mid = (start + end) / 2        radius = (end - start) / 2        theta = np.linspace(0, np.pi, 100)        x = mid + radius * np.cos(theta)        y = 3 + radius * np.sin(theta)         color = 'black' if link in special_crosslinks else arc_color        ax.plot(x, y, color=color, linewidth=arc_thickness)     # Tick marks and labels (1-based indexing)    tick_line_y = 0.8    for i in range(1, protein_length + 1):        if i % 10 == 0:            ax.plot([i, i], [0.9, tick_line_y], color='black', linewidth=1)            ax.text(i, tick_line_y - 0.1, f'{i}', ha='center', va='top', fontsize=8)        else:            ax.plot([i, i], [0.95, tick_line_y], color='black', linewidth=0.5)     ax.set_xlim(-5, protein_length + 5)    ax.set_ylim(0, max([abs(link[1] - link[0]) for link in crosslinks]) / 2 + 5)    ax.set_aspect('equal')    ax.axis('off')    plt.title("Protein Crosslinking Visualization")     if save_path:        plt.savefig(save_path, format='svg')     plt.show() # Example usageprotein_sequence = "MKYKPPKRYQPKNASWTTKRLYKYLEDKLEPKYDYKARVRAEKLVETIYHFTKEVKKHEVAPNDAVDVLKHEMARLDIVKTHFDFYQFFHDFMPREIRVKVVPDIVNKITIPRNGVFSEILSGHAVHA "  # Example amino acid sequencecrosslinks = [(32, 46), (110, 64), (30, 57), (56, 59), (104, 57), (100, 59), (32, 42), (27, 57), (28, 59), (52, 46), (30, 47), (26, 57), (30, 56), (108, 67), (27, 56), (28, 46), (49, 46), (28, 54), (30, 43), (110, 67), (30, 49), (26, 56), (27, 47), (56, 64), (32, 54), (47, 46), (100, 64), (24, 59), (26, 47), (28, 64), (36, 59), (35, 59), (28, 42), (43, 64), (27, 43), (108, 59), (100, 67), (122, 64), (30, 52), (33, 59), (28, 67), (27, 49), (110, 59), (128, 70), (24, 54), (30, 53), (128, 67), (32, 59), (24, 67), (119, 80), (128, 72), (56, 67), (26, 43), (128, 59), (24, 64), (128, 57), (26, 49), (122, 54), (128, 54), (118, 77), (128, 64), (104, 86), (64, 1), (59, 1), (24, 46), (122, 77), (128, 80), (128, 32), (100, 84), (122, 84), (59, 5), (64, 5), (27, 52), (128, 33), (96, 86), (128, 81), (128, 52), (128, 53), (27, 53), (128, 34), (118, 91), (122, 34), (67, 1), (26, 52), (26, 53), (128, 35), (128, 77), (110, 104), (122, 30), (128, 84), (128, 30), (67, 5), (24, 42), (43, 67), (108, 64)]  # Example crosslinksspecial_crosslinks = [(100,59), (100,64), (100,67), (100,84), (96,86) ]  # Example special crosslinks with different colorarc_color = 'darkgray'arc_thickness = 1.25save_path = '/Users/ssankara/Documents/bmT_180425_2.svg'  # Save path for the SVG file plot_crosslinked_protein(protein_sequence, crosslinks, special_crosslinks, arc_color='darkgray', arc_thickness=arc_thickness, save_path=save_path)
