# Supplementary material for: Insects evolved a monomeric histone-fold domain in the CENP-T protein family
Source: EMBO Rep. 2025 Oct 29;26(23):5799–825. doi: 10.1038/s44319-025-00603-5 (PMC12678787; doi:10.1038/s44319-025-00603-5)
Supplement: Supplementary file 10 — Expanded View Figures [file 44319_2025_603_MOESM10_ESM.pdf]

Expanded View Figures

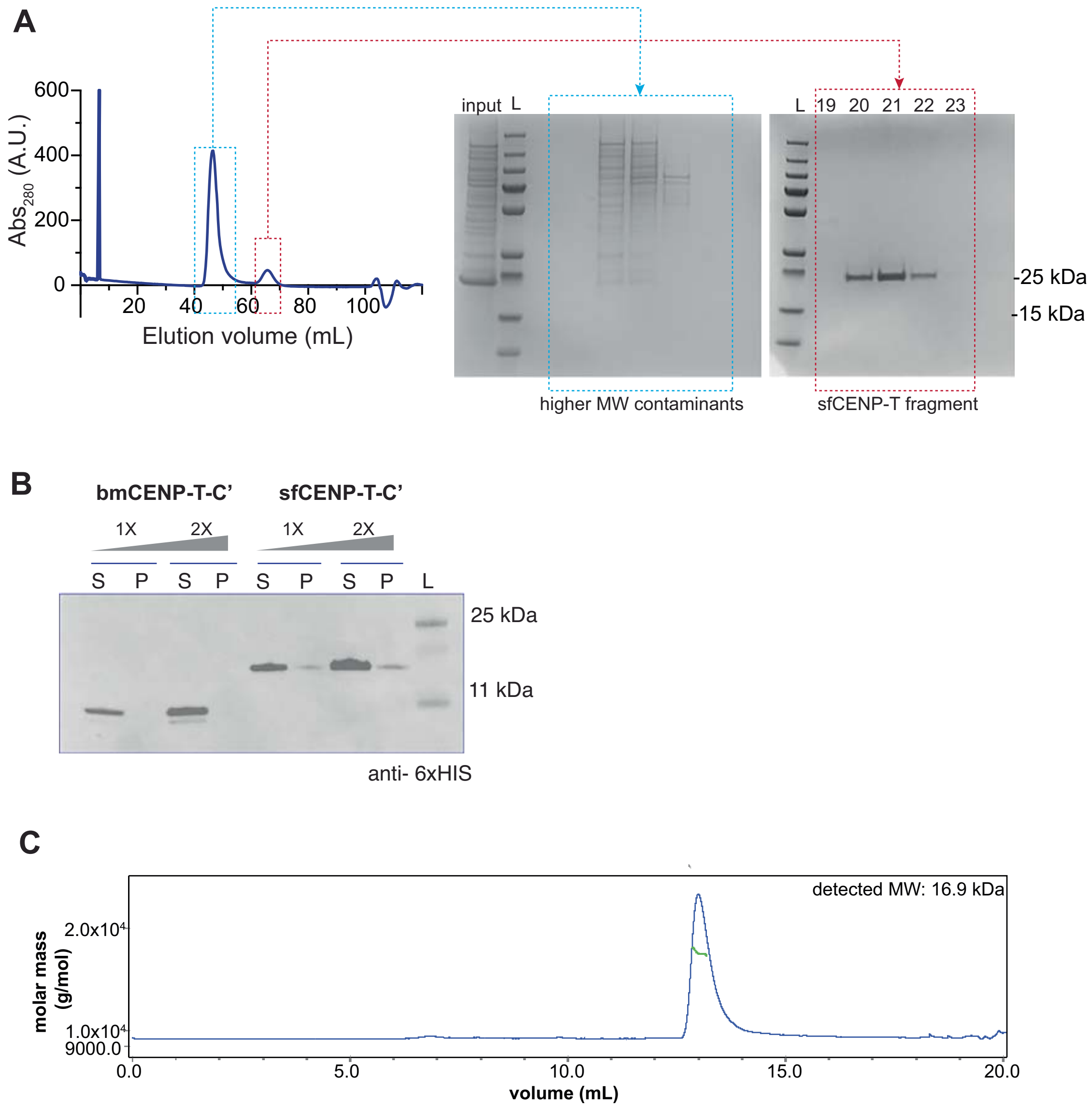

Figure EV1. Solubility of lepidopteran histone fold in SF9 and *E. coli* cells.

(**A**) An expanded view SEC profile and the corresponding SDS-PAGE analysis presented in Fig. 1C to highlight all the indicated fractions of 6xHis-sfCENP-T$^{1147\text{-}1314}$ purification. The SDS page profile of the peak in red box from Fig. 1C is reused in this panel to compare with the contaminating proteins eluted in the peak boxed in blue. Input: a fraction of the sample loaded into the SEC column, L: molecular weight marker. (**B**) Western blot analysis of the soluble (S) and pellet (P) fractions of *E. coli* BL21 cells expressing 6xHis-bmCENP-T (16.2 kDa) and 6xHis-sfCENP-T (21.5 kDa) fragment with anti-His antibodies. L: molecular weight marker. (**C**) SEC-MALS analysis of bmCENP-T$^{894\text{-}1016}$ fragment reveals the monomeric state of the protein. The molecular weight inferred from MALS (16.9 kDa) is represented as a blue line overlaid on the SEC peak for this protein. Source data are available online for this figure.

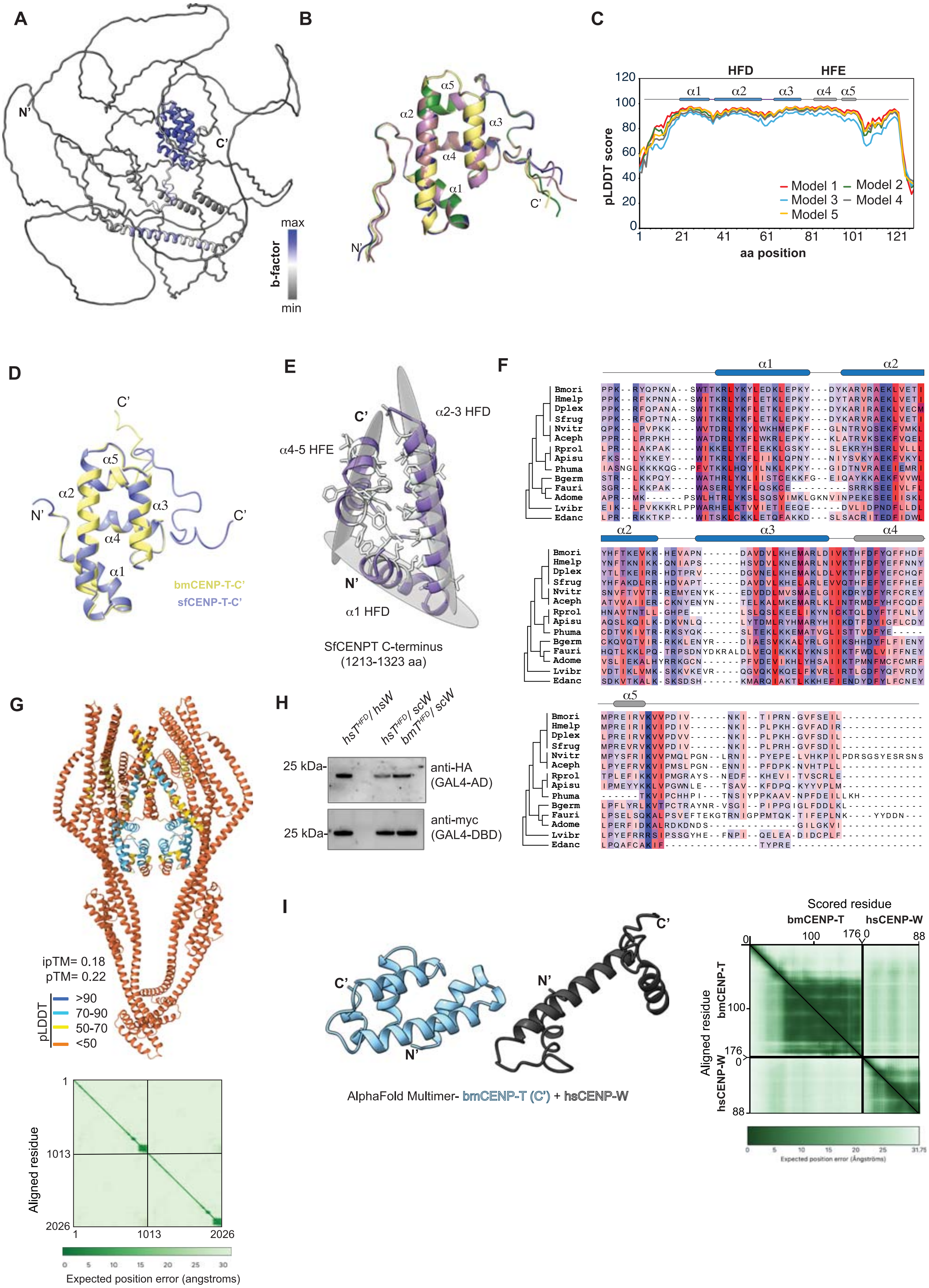
A
N'
C'
max
b-factor
min
B
α5
α2
α3
α4
α1
C'
N'
C
HFD
HFE
α1
α2
α3
α4
α5
120
100
80
60
40
20
0
pLDDT score
1
21
41
61
81
101
121
aa position
Model 1
Model 2
Model 3
Model 4
Model 5
D
C'
α5
α2
α3
N'
C'
α4
α1
bmCENP-T-C'
sfCENP-T-C'
E
C'
α2-3 HFD
α4-5 HFE
N'
α1 HFD
SfCENPT C-terminus
(1213-1323 aa)
F
α1
α2
α3
α4
α5
Bmori
Hmelp
Dplex
Sfrug
Nvitr
Aceph
Rprol
Apisu
Phuma
Bgerm
Fauri
Adome
Lvibr
Edanc
G
ipTM= 0.18
pTM= 0.22
pLDDT
>90
70-90
50-70
<50
1
1013
2026
Aligned residue
Expected position error (angstroms)
H
hsT^HFD / hsW
hsT^HFD / scW
bmT^HFD / scW
25 kDa-
anti-HA
(GAL4-AD)
25 kDa-
anti-myc
(GAL4-DBD)
I
C'
N'
C'
N'
AlphaFold Multimer- bmCENP-T (C') + hsCENP-W
Scored residue
bmCENP-T
hsCENP-W
0
100
176
0
88
Aligned residue
bmCENP-T
hsCENP-W
Expected position error (Ångströms)

◀ **Figure EV2. Structural basis for the stability of bmCENP-T in the absence of an interacting partner.**

(**A**) AlphaFold predicted structure of full-length bmCENP-T colored based on the confidence scores for each residue (bfactor-pLDDT score). (**B**) Overlay of the top five bmCENP-T models generated by AlphaFold. The helices of the HFD (α1–3) and the extension (α4-5) are indicated. The sequences upstream of the HFD in these models are hidden for clarity. (**C**) The pLDDT plot for the five models shown in (**A**). The regions corresponding to the helices of the HFD, and the extension are highlighted by a line diagram above the plot. (**D**) The best-ranked AlphaFold models for bmCENP-T and sfCENP-T are overlaid to highlight structural similarities at the C-terminus. Only the regions corresponding to the HFD and the HFE are shown in the panel. (**E**) Similarity in the stabilization of the monomeric HFD in sfCENP-T by the hydrophobic residues in the extension as shown for bmCENP-T in Fig. 2C. (**F**) Multiple sequence alignment of C-terminal fragment of CENP-T containing the HFD and HFE from several insect orders. The alignment was generated by MAFFT and was colored using the hydrophobicity scheme in JalView. The line diagram above the sequences indicates the helices of HFD (α1–3) and HFE (α4-5) as in *B. mori*. The cladogram represents insect orders where CENP-T has been detected. The sequences species and the orders they represent are as follows: Lepidoptera (*B. mori, Heliconius melpomene, Danaus plexippus, S. frugiperda*), Hymenoptera (*Nasonia vitripennis*, *Atta cephalotes*), Hemiptera (*Rhodnius prolixus, Acyrthosiphon pisum*), Phthiraptera (*Pediculus humanus corporis*), Blattoeda (*Blatella germanica*), Dermaptera (*Forficula auricularia)*, Orthoptera *(Acheta domestica*), Odonata (*Libuellua vibrans*), Ephemeroptera (*Ephemera dancia*). (**G**) The AlphaFold3 model of a homodimeric full-length bmCENP-T colored based on pLDDT scores. The pTM and ipTM values generated by the AlphaFold webserver are indicated. The predicted aligned error matrix is presented below the model. It should be noted that, unlike the prediction for bmCENP-T monomer, the N-terminus of CENP-T is predominantly helical in the homodimer, albeit with a low complex confidence score. (**H**) Whole cell extracts from cells used in the spot dilution assay shown in Fig. 2G were analyzed by immunoblotting using anti-Myc and anti-HA antibodies. (**I**) The inability of a monomeric HFD (from bmCENP-T) to interact with a canonical HFD (from hsCENP-W) as revealed by AlphaFold multimer. The predicted aligned error (PAE) matrix is presented on the right. Source data are available online for this figure.

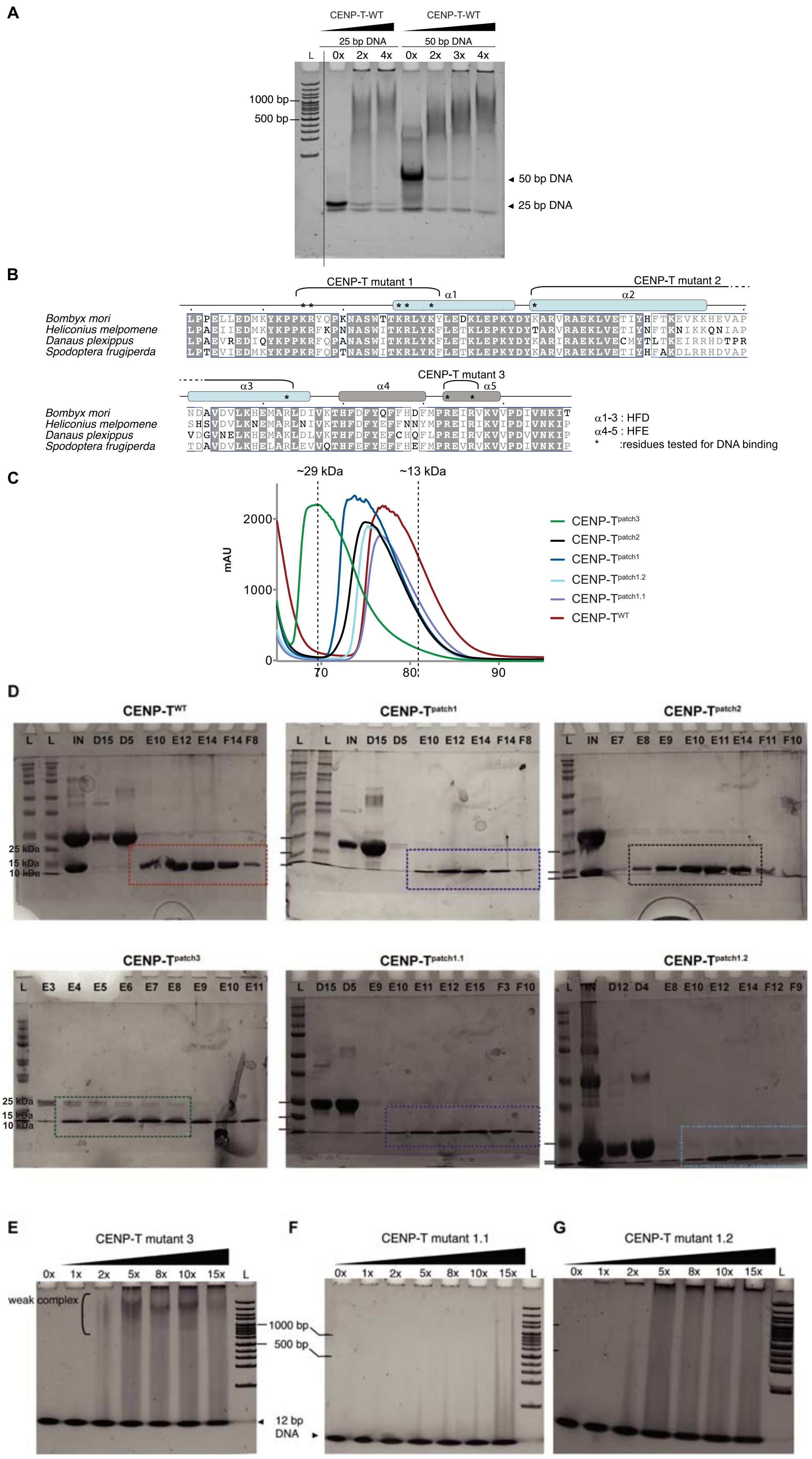
A
CENP-T-WT
CENP-T-WT
25 bp DNA
50 bp DNA
L 0x 2x 4x 0x 2x 3x 4x
1000 bp
500 bp
50 bp DNA
25 bp DNA
B
CENP-T mutant 1
CENP-T mutant 2
CENP-T mutant 3
Bombyx mori
Heliconius melpomene
Danaus plexippus
Spodoptera frugiperda
α1-3 : HFD
α4-5 : HFE
* :residues tested for DNA binding
C
~29 kDa
~13 kDa
mAU
CENP-Tpatch3
CENP-Tpatch2
CENP-Tpatch1
CENP-Tpatch1.2
CENP-Tpatch1.1
CENP-TWT
D
CENP-TWT
CENP-Tpatch1
CENP-Tpatch2
CENP-Tpatch3
CENP-Tpatch1.1
CENP-Tpatch1.2
25 kDa
15 kDa
10 kDa
E
CENP-T mutant 3
weak complex
F
CENP-T mutant 1.1
G
CENP-T mutant 1.2
0x 1x 2x 5x 8x 10x 15x L
1000 bp
500 bp
12 bp DNA

 © The Author(s)

Figure EV3. Residues in patch 2 and patch 3 are dispensable for DNA binding.

(**A**) Native PAGE gels of DNA titration using different molar ratios of bmCENP-$T^{894-1016}$-WT and a 25 bp or a 50 bp DNA fragment at 100 mM NaCl. Lane L: 100 bp DNA marker. (**B**) Multiple sequence alignment of the C′ fragment of CENP-T between 4 lepidopteran species. Identical residues are highlighted with a gray background. The residues tested for DNA binding function are marked with an asterisk. (**C**) The SEC elution profile of the WT and mutant versions of bmCENP-T used in the DNA binding assays in Figs. 3 and EV3. (**D**) The Coomassie stained SDS-polyacrylamide gels containing the SEC fractions for the purification of the indicated versions of bmCENP-T after cleaving the GST tag. The quality of each sample studied in S3C (colored boxes) is visually represented. L- Ladder, IN- Input sample, alphanumeric labels starting with D/E/F are different fractions collected in the experiment. (**E–G**) Native PAGE gels of DNA titration using different molar ratios of bm CENP-$T^{894-1016}$ -patch 3 mutant (**C**), bmCENP-$T^{894-1016}$ patch 1.1 mutant with the substitutions K895A, R896A, and K907A (**D**) and bmCENP-$T^{894-1016}$ patch 1.2 mutant with the substitutions R908S and K911A (**E**). Lane L: 100 bp DNA marker.

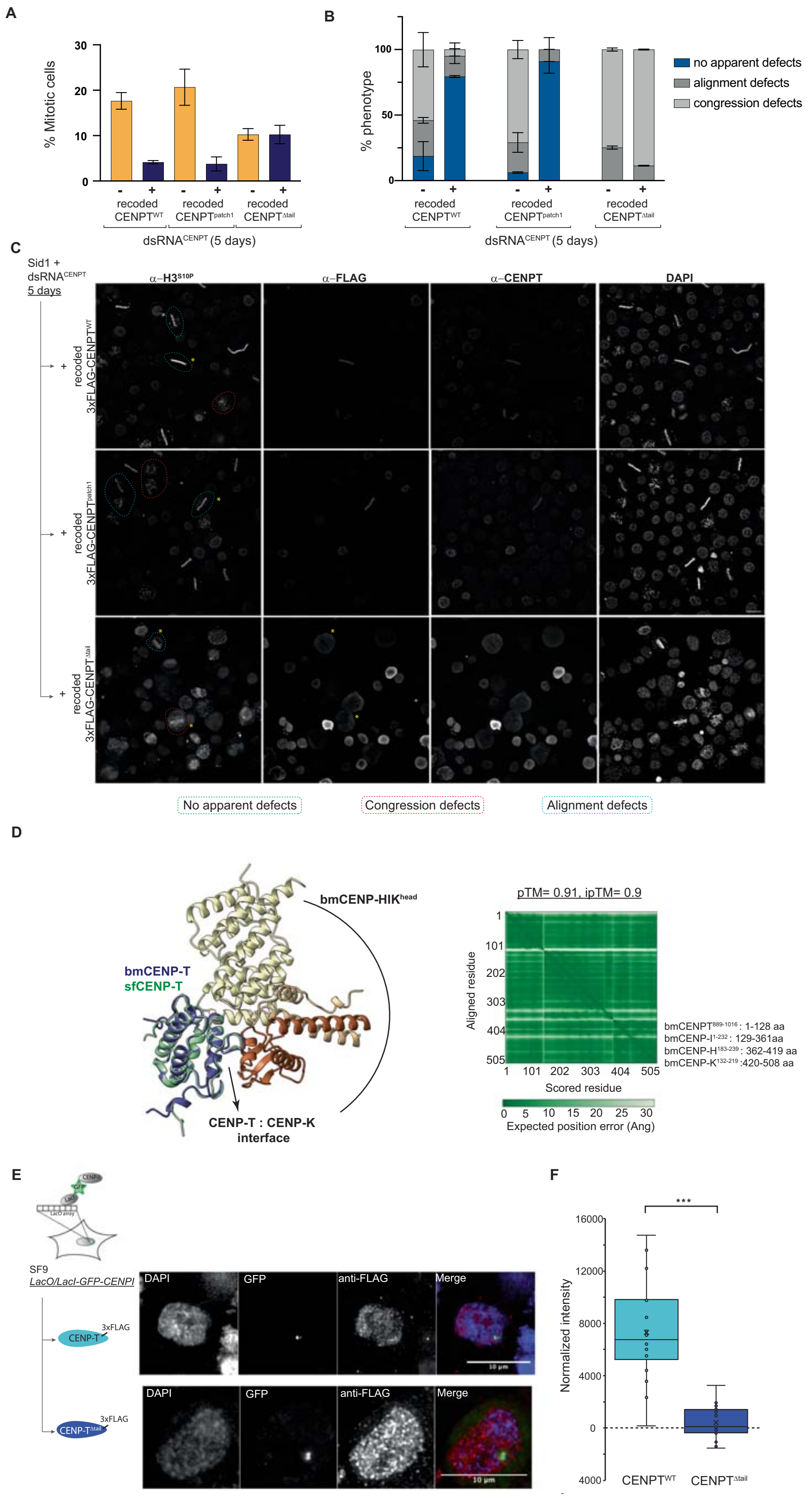

A
% Mitotic cells
30
20
10
0
- + - + - +
recoded CENPT^WT
recoded CENPT^patch1
recoded CENPT^Δtail
dsRNA^CENPT (5 days)
B
% phenotype
100
50
0
no apparent defects
alignment defects
congression defects
C
Sid1 + dsRNA^CENPT 5 days
α–H3^S10P
α–FLAG
α–CENPT
DAPI
recoded 3xFLAG-CENPT^WT
recoded 3xFLAG-CENPT^patch1
recoded 3xFLAG-CENPT^Δtail
No apparent defects
Congression defects
Alignment defects
D
bmCENP-HIK^head
bmCENP-T
sfCENP-T
CENP-T : CENP-K interface
pTM= 0.91, ipTM= 0.9
Aligned residue
Scored residue
1 101 202 303 404 505
bmCENPT^889-1016 : 1-128 aa
bmCENP-I^1-232 : 129-361aa
bmCENP-H^183-239 : 362-419 aa
bmCENP-K^132-219 :420-508 aa
0 5 10 15 20 25 30
Expected position error (Ang)
E
SF9
LacO/LacI-GFP-CENPI
LacO array
DAPI
GFP
anti-FLAG
Merge
3xFLAG
CENP-T
CENP-T^Δtail
10 µm
F
***
Normalized intensity
16000
12000
8000
4000
0
4000
CENPT^WT
CENPT^Δtail

 © The Author(s)

Figure EV4. Properties and roles of the *B. mori* CENP-T as part of the kinetochore in *B. mori* cells.

(**A**) Mitotic index of untransfected (gray) or transfected (blue) cells expressing RNAi-resistant versions of bmCENP-T^{WT}, bmCENP-T^{patch1}, or bmCENP-$T^{\Delta tail}$ upon depletion of endogenous CENP-T. (**B**) Fraction of cells with or without mitotic defects, and the nature of defects observed (congression or alignment defects) upon endogenous CENP-T depletion in lines expressing bmCENP-T^{WT}, bmCENP-T^{patch1}, or bmCENP-$T^{\Delta tail}$. (**C**) Representative examples of untransfected or transfected cells expressing bmCENP-T^{WT}, bmCENP-T^{patch1}, or bmCENP-$T^{\Delta tail}$ in mitosis stained against phospho histone H3 (anti-$H3^{S10P}$), anti-FLAG, anti-CENP-T, and DAPI. Scale bar, 10 µm. (**D**) AlphaFold multimer model of the interaction between bmCENP-$T^{889\text{-}1016}$ and the bmCENP-T-HIK^{head} constituted by bmCENP-T-$I^{1\text{-}232}$, bmCENP-T-$H^{183\text{-}239}$, and bmCENP-T-$K^{132\text{-}219}$. The confidence scores and the predicted aligned error matrix are presented on the right. The AlphaFold model of full-length sfCENP-T was superimposed over the CENP-HIK^{head}:T model. The disordered N terminus of sfCENP-T upstream of the histone fold were hidden for clarity. (**E**) Representative images of DAPI-stained cells showing localization pattern of 3xFLAG-tagged sfCENP-T^{WT} (top) or sfCENP-$T^{\Delta tail}$ (bottom) in Sf9-LacO lines transiently expressing LacI-GFP-sfCENPI constructs. Merge: DNA (blue), expressing LacI-GFP-sfCENPI (green) and 3xFLAG-tagged sfCENP-T^{WT} (top) or sfCENP-$T^{\Delta tail}$ (red). Scale bar: 10 µm. (**F**) Quantifications of normalized mean fluorescence intensity of 3xFLAG-tagged sfCENP-T^{WT} or sfCENP-$T^{\Delta tail}$ at the LacI foci. Statistical significance was tested using the Student's *t* test (*** indicates $P = 3.8 \times 10^{-7}$). The edges of the box mark the 25th and 75th percentile and the line inside marks the median value. Source data are available online for this figure.

© The Author(s)

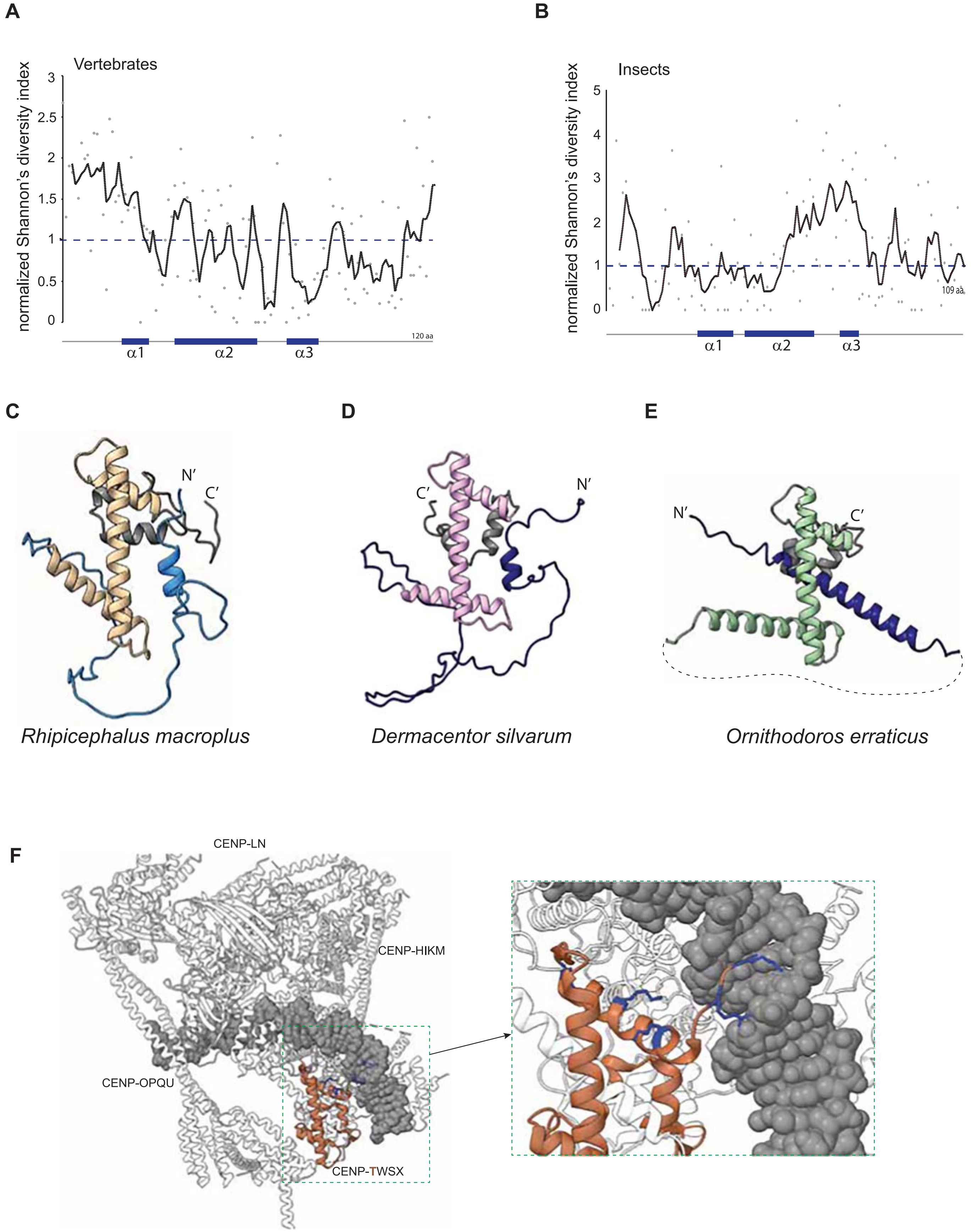
A
B
Vertebrates
normalized Shannon's diversity index
3
2.5
2
1.5
1
0.5
0
120 aa
α1
α2
α3
Insects
normalized Shannon's diversity index
5
4
3
2
1
0
109 aa
α1
α2
α3
C
D
E
N′
C′
C′
N′
N′
C′
Rhipicephalus macroplus
Dermacentor silvarum
Ornithodoros erraticus
F
CENP-LN
CENP-HIKM
CENP-OPQU
CENP-TWSX

Figure EV5. Diversity in the sequence and structure of the monomeric HFD in Arthropoda.

(**A**, **B**) Comparative analysis of sequence diversity in vertebrate and insect CENP-T using Shannon's entropy analysis. The gray dots represent the normalized Shannon's diversity ratio for each amino acid in the C′ of CENP-T. The trendline represents the moving average of 5 datapoints. (**C–E**) The AlphaFold model for CENP-T from three acariformes, in which CENP-W remains undetected. The canonical HFD structure in each of the three species is highlighted in beige, pink, and green, respectively. The HFE is colored gray and a part of the N′ of the protein that blocks the interaction space for CENP-W is shaded blue. For protein IDs see Dataset EV2. (**F**) The structure of human CCAN reported in Yatskevitch et al (2022) highlighting the proximity of α1 of CENP-T HFD (CENP-T is colored brown) to DNA (gray). The side chains of the positively charged amino acids in this region are shown in blue. The illustration was adapted from PDB 7R5S.
